# Supplementary material for: Maternal opioids downregulate adiponectin receptor signaling and alter growth in offspring: pilot study
Source: Front Pediatr. 2026 Apr 1;14:1755488. doi: 10.3389/fped.2026.1755488 (PMC13079689; doi:10.3389/fped.2026.1755488)
Supplement: Supplementary file 1 [file Table1.docx]

**Supplemental Table S1. Gene expression by exposure and treatment**

|  | ***ADIPOR1* ΔCt *^a^*** |
| --- | --- |
| **Non-Exposed (N=32)** | 1.79 (1.47) |
| **Opioid-Exposed (N=35)** | 3.66 (3.30) |
| **Exposed Tx (n=28)** | 3.04 (2.19) |
| **Exposed No Tx (n=7)** | 6.13 (5.60) |

^a^ Data are presented as mean (standard deviation). Tx: pharmacotherapy requirement.

ΔCt values are inversely proportional to the gene expression levels.
