# Supplementary material for: Maternal opioids downregulate adiponectin receptor signaling and alter growth in offspring: pilot study
Source: Front Pediatr. 2026 Apr 1;14:1755488. doi: 10.3389/fped.2026.1755488 (PMC13079689; doi:10.3389/fped.2026.1755488)
Supplement: Supplementary file 2 [file Table2.docx]

**Supplemental Table S2. Gene expression by sex and exposure**

|  | **FEMALES (N=38)** | | | **MALES (N=27)** | | | **Difference in opioid effect by sex (95% CI)** ^c^ |
| --- | --- | --- | --- | --- | --- | --- | --- |
|  | **Non-Exposed**  **(n=19)** ^a^ | **Opioid-Exposed (n=19)** ^a^ | **Difference (95% CI)** ^b^ | **Non-Exposed**  **(n=13)** ^a^ | **Opioid-Exposed (n=16)** ^a^ | **Difference (95% CI)** ^b^ |  |
| ***ADIPOR1 ΔCt*** | 2.04 (1.51) | 4.48 (4.11) | 2.44 (0.41, 4.48) | 1.32 (1.35) | 2.67 (1.60) | 1.36 (0.21, 2.50) | -1.09 (-3.59, 1.42) |

^a^ Data are presented as mean (standard deviation). ^b^ Data are presented as mean difference (confidence interval) from a linear regression model restricted to females or males. ^c^ Data are presented as mean difference (confidence interval) from a linear regression model that includes an interaction term between exposure and sex. ΔCt: delta threshold cycle, values are inversely proportional to the gene expression levels
